# Role of autophagy in ischemic stroke: insights from animal models and preliminary evidence in the human disease

**DOI:** 10.3389/fcell.2024.1360014

**Published:** 2024-03-25

**Authors:** Rosita Stanzione, Donatella Pietrangelo, Maria Cotugno, Maurizio Forte, Speranza Rubattu

**Affiliations:** ^1^ IRCCS Neuromed, Pozzilli, Italy; ^2^ Clinical and Molecular Medicine Department, School of Medicine and Psychology, Sapienza University of Rome, Rome, Italy

**Keywords:** ischemic stroke, autophagy, mitochondria, mTOR, animal models, human disease

## Abstract

Stroke represents a main cause of death and permanent disability worldwide. The molecular mechanisms underlying cerebral injury in response to the ischemic insults are not completely understood. In this article, we summarize recent evidence regarding the role of autophagy in the pathogenesis of ischemic stroke by reviewing data obtained in murine models of either transient or permanent middle cerebral artery occlusion, and in the stroke-prone spontaneously hypertensive rat. Few preliminary observational studies investigating the role of autophagy in subjects at high cerebrovascular risk and in cohorts of stroke patients were also reviewed. Autophagy plays a dual role in neuronal and vascular cells by exerting both protective and detrimental effects depending on its level, duration of stress and type of cells involved. Protective autophagy exerts adaptive mechanisms which reduce neuronal loss and promote survival. On the other hand, excessive activation of autophagy leads to neuronal cell death and increases brain injury. In conclusion, the evidence reviewed suggests that a proper manipulation of autophagy may represent an interesting strategy to either prevent or reduce brain ischemic injury.

## 1 Introduction

Stroke ranks as the second leading cause of mortality on a global scale, standing at the third place in the developed countries following cardiovascular diseases and cancer. Stroke accounts for 10%–12% of all annual deaths (Favate and Younger, 2016; Benjamin et al., 2019) and it also represents the first cause of disability and the second cause of dementia (Williams et al., 2019). Ischemic stroke (IS), the predominant form of stroke (about 87% occurrence), is caused by an occlusion of either a large or a small cerebral blood vessel whereas a rupture of an artery leads to the less frequent hemorrhagic type of stroke. The interruption of blood flow compromises the main physiological processes of cerebral cells, and leads to an increase of calcium overload, inflammatory responses, oxidative stress, alteration of the blood–brain barrier (BBB) permeability, and excitotoxity. The inflammatory response and oxidative stress are among the earliest events that characterize the cascade of cerebral ischemic injury. They cause the dysfunction of several neural mechanisms, such as the antioxidant defense system. They also increase the expression of the pro-inflammatory nuclear factor (NF)-kB and inhibit the synthesis of anti-inflammatory proteins such as cAMP responsive element binding protein (CREB) and activator protein 1 (AP-1) (Rana and Singh, 2018).

It has been reported that a dysregulation of autophagy may contribute to worsening brain damage (George and Steinberg, 2015; Wang et al., 2018; Shi et al., 2023). Autophagy is an intracellular mechanism by which cells remove damaged or senescent cytoplasmic components. Autophagy helps to maintain cellular homeostasis by eliminating dysfunctional organelles, misfolded proteins as well as other cellular debris (Gomez-Virgilio et al., 2022). The role of autophagy has been clearly identified in several diseases, particularly in the context of neurodegenerative and cardiovascular diseases, cancers, inflammatory disorders, and autoimmune diseases. Mutations of autophagy-related genes are currently recognized as causative factors in Mendelian disorders (Grosjean et al., 2022). Polymorphisms in autophagy related genes were associated with increased vulnerability to specific pathologic conditions (Grosjean et al., 2022). Accumulating evidence demonstrates that the increase of inflammation and oxidative stress, following IS, can induce the development of the autophagic process which, in turn, may exert both protective and detrimental effects, depending on the type of stress and on its duration, and on the type of cells involved. A moderate level of autophagy protects neurons and restores cerebral function by reducing the inflammatory process and oxidative stress, whereas excessive autophagy induces neuronal cell death and exacerbates brain damage (Hu et al., 2015; He et al., 2021). Therefore, the exact role of autophagy in the pathogenesis of IS has not being completely understood. In this article we provide an overview of the relevant literature regarding the involvement of autophagy in IS both in preclinical models and in humans, also highlighting gaps in knowledge.

## 2 Overview of autophagy biology

Autophagy plays a crucial role in maintaining tissue homeostasis, and it is generally activated in response to various stresses or nutrients deprivation to provide energy and necessary elements for cell survival (Anderson and Macleod, 2019). In some circumstances, autophagy provides energy accumulation needed for later use and, at the same time, recycles macromolecules and eliminates harmful material (Klionsky, 2005). According to the mode of cargo delivery to lysosomes, three different forms of autophagy are described: macroautophagy, microautophagy and chaperone-mediated autophagy (CMA) (Wang et al., 2018; Yamamoto and Matsui, 2023). Macroautophagy is the lysosome-mediated degradation of cytoplasmic cargo in the autophagosome and involves four phases: i) the formation of an incomplete double-membrane vesicle termed phagophore; ii) the elongation phase in which the phagophore maturates into the autophagosome, able to sequester the cytoplasmic cargo; iii) a fusion phase in which the autophagosome merges with the lysosome and leads to autophagolysosome formation; and iiii) a degradation phase in which the cargo is degraded to essential elements which are then recycled and reintroduced into cell metabolism (Griffey and Yamamoto, 2022). All four phases require the involvement of several autophagy related (ATG) proteins. The family of ATG proteins includes 16–20 members that share a highly homology in mammals. ATG proteins are classified in six functional groups: 1) the unc-51 like autophagy activating kinase 1 (ULK1)-ATG13-FIP200-ATG101 protein kinase complex involved in the initiation of autophagosome formation through direct interaction with Atg16L1; 2) the class III phosphatidylinositol 3-kinase (PtdIns3K) complex, containing the vacuolar protein sorting 34 homolog (VPS34), VPS15 and Beclin one proteins, that plays a fundamental role in the nucleation and maturation of the phagophore; 3) the WD repeat domain, phosphoinositide interacting (WIPI)/ATG18-ATG2 complex that participates in the initial phase of membrane elongation, in the interaction between autophagosomes and the endoplasmic reticulum (ER) and in the lipid transport; 4) the multi-spanning transmembrane protein ATG9A involved in the autophagosome membrane expansion; 5) the ubiquitin-like ATG5/ATG12 system and 6) the ubiquitin-like ATG8/microtubule-associated proteins 1A/1B light chain 3B (LC3) conjugation system that also contributes to autophagosome membrane formation and elongation (Nishimura et al., 2013; Wesselborg and Stork, 2015; Graef, 2018; Levine and Kroemer, 2019; Matoba et al., 2020).

Microautophagy is a non-selective lysosomal degradative process. It involves direct swallow of cytoplasmic cargo at border membrane by autophagic tubes, which mediate both invagination and vesicle scission into the lumen (Li et al., 2012). In the CMA, the substrates to be removed are labeled with a KFERQ motif and later complexed to chaperons, as heat shock proteins (HSPs), for the subsequent delivery to lysosomes mediated by lysosomal-associated membrane protein 2 (LAMP2) (Cuervo and Wong, 2014).

## 3 Role of autophagy in human diseases

Several evidence demonstrated that defective autophagy contributes to neurodegenerative diseases (Pickford et al., 2008; Klionsky et al., 2021). In this regard, mutations in ATG genes were found in patients with neurodegenerative diseases, reflecting in the accumulation of toxic aggregates which represent a common determinant of these pathological conditions (Klionsky et al., 2021). Pharmacological reactivation of autophagy was reported to exert protective effects in preclinical models of neurodegenerative diseases, such as Huntington, Alzheimer, prion disease, spinocerebellar ataxia type 3, and Parkinson (Nixon, 2013). Autophagy is also fundamental in the response to the most common infections and in the onset of autoimmune diseases (Deretic et al., 2013; Yang et al., 2015). Defects in autophagy affect the generation, survival, maturation and properties of cellular components of both innate and adaptive immunity systems (Ma et al., 2013).

In cancer, autophagy plays a dual role since it can promote both survival and death of tumor cells, depending on the cancer type and stage (Chen et al., 2021; Debnath et al., 2023). Other reports suggest a fundamental role of autophagy in metabolic disorders with a tissue-specific role in liver and adipose tissue (Menikdiwela et al., 2020). Several reports also highlighted the importance of autophagy in the pathophysiology of cardiovascular diseases such as atherosclerosis, cardiac ischemia, and stroke (Sciarretta et al., 2018; Wang et al., 2021). In this regard, Liao et al. demonstrated that autophagy plays an important role in reducing inflammasome-activation by macrophages in atherosclerotic lesions. The authors showed the induction of autophagy and autophagic flux in primary murine macrophages treated with 7-ketocholesterol, a bioactive sterol able to promote apoptosis and oxidative stress in macrophages. The autophagy activation diminished macrophage apoptosis, consequently mitigating the pathogenesis and progression of atherosclerosis, whereas the inhibition of autophagy, through ATG5 silencing, promoted apoptosis and oxidative stress (Liao et al., 2012). Autophagy limits cardiac injury in mouse models of heart failure and metabolic cardiomyopathy (Sciarretta et al., 2018). Of interest, autophagy has been demonstrated to exert both protective and detrimental effects in response to myocardial ischemia/reperfusion. In this regard, autophagy activation before ischemia reduces I/R injury whereas high levels of autophagy during reperfusion exacerbate I/R injury. At the molecular level, excessive autophagy during reperfusion leads to a form of cell death termed autosis (Sciarretta et al., 2018).

## 4 Role of autophagy in stroke

Over the last decades, autophagy activation emerged as a potential therapeutic and preventive strategy toward stroke in preclinical models (Forte et al., 2020; Forte et al., 2021a; Forte et al., 2021b; Wang et al., 2021). In regard to stroke, autophagy is activated in response to IS in all brain cells present in the damaged area such as neurons, glial cells, and microvascular cells (Perez-Alvarez et al., 2018). The current concept regarding the role of autophagy in IS that it exerts either protective or deleterious effects, depending on the duration of stress (permanent or transient), and it also differs in the ischemic vs. the reperfusion phase. Moreover, the dual role of autophagy depends on the cell type involved (Wang et al., 2021). Generally, at the neuronal level, a moderate degree of autophagy exerts protective effects because it ensures the elimination of protein aggregates, as also observed in neurodegenerative disorders including the Alzheimer’s disease (Balin et al., 2011; Medeiros et al., 2011). However, a defective or excessive autophagy can lead to neuronal cell death. In fact, an excessive level of autophagy in neonatal mice hippocampal pyramidal neurons under hypoxia/ischemia (H/I) produces, by both caspase-dependent and-independent mechanisms, important nuclear alterations resulting in neuronal injury (Chu, 2008; Uchiyama et al., 2008).

### 4.1 Mechanisms regulating autophagy in IS

**FIGURE 1:**
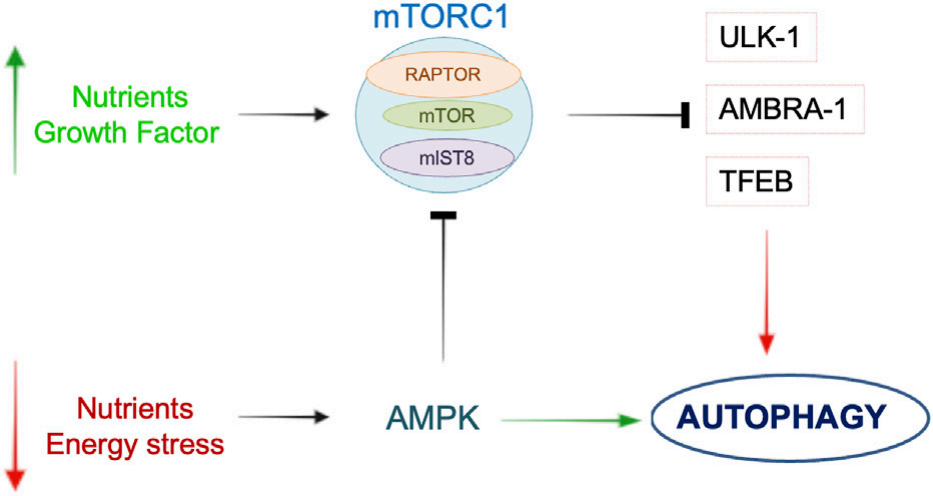
Schematic overview of the main pathways involved in the regulation of autophagy. In the presence of nutrients or growth factors, mTORC1 is activated and it inhibits autophagy by acting on ULK-1, AMBRA-1 or TFEB. During nutrient or energy deprivation, AMPK promotes autophagy through either direct activation of autophagic proteins or mTORC1 inhibition. See text for further details.

In models of IS autophagy is mostly regulated by the mechanistic target of rapamycin (mTOR) complex I (mTORC1) and by AMPK (Adenosine monophosphate-activated protein kinase (Figure 1), which interact with multiple signaling including the Phosphoinositide-3-kinase (PI3K)/Protein Kinase B (Akt), Mitogen-activated protein kinase (MAPK), NF-κB, Tumor protein p53, and B-cell lymphoma 2 (Bcl2) pathways (Wang et al., 2021). In the specific, mTOR is an atypical serine/threonine kinase and the core component of two separate protein complexes named mTORC1 and mTOR complex 2 (mTORC2). According to the bioavailability of nutrients, growth factors, energy and oxygen, mTORC1 promotes anabolic processes and inhibits catabolic mechanisms, such as autophagy (Laplante and Sabatini, 2013). The PI3K/Akt mediates mTOR activation through the inhibition of tuberous sclerosis complexes (TSC-1/2). The modulation of the PI3K/Akt/mTOR improved neuronal survival both *in vivo* and *in vitro* in IS (Wei et al., 2016). Extracellular signal-regulated kinase (ERK), a member of MAPK family, regulates autophagy in IS through the inhibition of mTORC1 (Xu et al., 2021).

The AMPK pathway receives both activator and inhibitory inputs from stress sensors (Marino et al., 2014). In the brain, AMPK activity increases following an ischemic insult, due to the reduction of the adenosine triphosphate (ATP)/adenosine monophosphate (AMP) ratio, to stimulate protective autophagy by inhibition of mTORC1 (Dai et al., 2017). Moreover, mTOR inhibition by AMPK occurs through the activation of TSC-1/2 (Garcia and Shaw, 2017). Another report demonstrated that AMPK activity is regulated by calcium level through calmodulin-dependent protein kinase β in rat brains (CaMKKβ) (Li et al., 2019). NF-kB, a regulator of inflammation and apoptosis, is an additional regulator of mTOR activity in mice undergoing cerebral ischemia. In this regard, NF-kB knockout mice showed an increased cerebral injury due to the activation of detrimental autophagy (Li et al., 2013). The latter mechanism may underline the interplay between apoptosis, inflammation, and autophagy during IS. mTORC1 also inhibits autophagy by phosphorylating the transcription-factor-EB (TFEB), a positive regulator of autophagy and transcriptional regulator of genes involved both in autophagosome formation and lysosomal biogenesis. However, TFEB modulation in models of IS not dependent by mTORC1 activation (Liu et al., 2019).

Another pathway that contributes to autophagy modulation during IS mediated by Beclin-1/B-Cell Leukemia/Lymphoma 2 (Bcl-2). Beclin one is an important protein involved in the initial phase of autophagosome formation. Beclin one expression increases in neurons in response to ischemia (Rami, 2008). In addition, Beclin one interacts with the antiapoptotic protein Bcl-2 to form a Beclin-1/Bcl-2 complex which inhibits autophagy. In a rat model of cerebral ischemia followed by reperfusion, remote ischemic conditioning induces Bcl-2 dissociation from Beclin 1, leading to the enhancement of autophagy and a consequent reduction of brain injury (Qi et al., 2015). However, other reports demonstrated that ischemia/reperfusion induces autophagy via endoplasmic reticulum stress (ER) induced inhibition of Bcl-2 (Feng et al., 2017). Therefore, the exact role of the Beclin 1/Bcl-2 signaling pathway in the pathophysiology of IS requires further investigation. Other evidence suggests that Rab7, a small GTPase, mediates the protective effects of hypoxic preconditioning in rats by promoting autophagosome formation (Zhan et al., 2017). Mitophagy, the selective form of autophagy devoted to the clearance of damaged mitochondria, also plays a role during cerebral ischemia/reperfusion. In this regard, the PTEN-induced kinase 1 (PINK1)/Parkin dependent mitophagy is activated during reperfusion in the brain of rat models of IS, due to the recruitment to mitochondria of Dynamin related protein (Drp1), a protein involved in mitochondrial fission mechanism (Feng et al., 2018).

## 5 Protective effect of autophagy in animal models of IS

Suitable animal models of IS include rats and mice subjected to either transient or permanent middle cerebral artery occlusion/reperfusion (MCAO) (Zeng et al., 2023). In a rat model of MCAO, Wang et al. demonstrated that overexpression of nicotinamide phosphoribosyl transferase (NAMPT), the rate-limiting enzyme in NAD^+^ biosynthesis, decreased the size of cerebral infarction via autophagy activation in an early stage of ischemia (2 h) and not after 8 and 24 h following ischemia (Wang et al., 2012). NAMPT increases NAD^+^ levels, which in turn contributes to activate Sirtuin-1 (SIRT1). Once activated, SIRT1 induces autophagy via inhibition of mTORC1. Of interest, another study further demonstrated that NAMPT is released in exosomes by astrocytes undergoing acute ischemia. Once released, NAMPT can activate autophagy in neurons, therefore improving the neurofunctional recovery (Deng et al., 2022). Recently, in mice undergoing MCAO, autophagy inhibition with 3-Methyladenine (3-MA) was shown to exacerbate brain injury. Mechanistically, overexpression of BAG3 (B-cell lymphoma 2-associated-athanogene 3), a protein involved in cellular protein quality control, activates autophagy and prevents both apoptosis and cerebral injury (Liu et al., 2023). Another study demonstrated that CAPN1 (calpain1), an intracellular Ca^2+^-regulated cysteine protease, is activated in the cortex of rats undergoing permanent MCAO. The latter leads to inhibition of the autophagic flux by impairing both lysosome function and autophagosome formation. CAPN1 inhibition rescues autophagic flux and reduces ischemic injury. The protective effects of CAPN1 inhibition are blunted by concomitant inhibition of autophagy by chloroquine (CQ) or 3-MA (Liu et al., 2021). In rats subjected to permanent MCAO, autophagy is upregulated in the early phase of ischemia (Liu et al., 2019), along with an increased activity of TFEB, especially in neurons of the cortex. At later stages after ischemia, nuclear translocation of TFEB decreases, resulting in the accumulation of autophagosomes and autophagy substrates, with a consequent exacerbation of the ischemic injury. The decreased activity of TFEB in the later phases of ischemia was not dependent by mTORC1 activation, which is a TFEB inhibitor. TFEB overexpression in neurons reduces ischemic damage. These results suggest that TFEB plays a pivotal role in the MCAO-mediated dysfunction of autophagy-lysosomal pathway.

Of interest, autophagy activation in the MCAO mouse model was also found to be regulated by non-coding RNAs, such as circular RNA (circ-RNA). In this regard, circ-RNA of FoxO3 (circ-FoxO3) improved BBB integrity via autophagy stimulation in mice undergoing MCAO for 2 h and being analyzed 2 h after reperfusion. At the molecular level, circ-FOXO3 stimulated autophagy via inhibition of mTORC1 (Yang et al., 2022). Other circ-RNAs were found to be modulated in models of MCAO and to play a role in autophagy regulation during IS (Mehta et al., 2017; Li et al., 2022). In this regard, circ-SHOC2 overexpression was reported to attenuate brain injury in mice undergoing MCAO through autophagy activation. Mechanistically, circ-SHOC2 sponges miR-7670-3p which regulates SIRT1 expression (Chen et al., 2020).

Carloni et al. showed that activation of autophagy by rapamycin, a mTORC1 inhibitor, was able to reduce the necrotic death of hippocampal and cortical neurons and to decrease brain injury in a neonatal mouse model of hypoxic-ischemic brain damage (Carloni et al., 2008). In a separate study, rapamycin reduced the infarct volume and improved neurological functions in rats undergoing focal MCAO (Wu et al., 2018). Of interest, mTOR was reported to be activated in the ischemic penumbra whereas it was inhibited in the ischemic core following MCAO. Some studies also demonstrated that the protective effects of rapamycin in the reduction of brain injury in transient MCAO rats associated with the activation of mitophagy (Li et al., 2014). Autophagy reactivation in transient MCAO models by ezetimibe, an inhibitor of NPC1L1, reduced neuronal damage and activated autophagy through an AMPK-dependent mechanism at 24 and 72 h following MCAO. The autophagy activation by ezetimibe also led to reduction of neuronal apoptosis (Yu et al., 2018). Another study demonstrated that cerebral ischemia increased TSC1 activity in cultures of rat hippocampal CA3 neurons with a consequent activation of autophagy, along with the improvement of cell survival. Upregulation of TSC1 protected CA3 neurons toward ischemia also in rats undergoing global forebrain ischemia (a more severe type of IS) (Papadakis et al., 2013). The latter was mediated by the inhibition of mTORC1 activity.

The administration of the natural flavonol kaempferol to cultures of murine neuronal cells undergoing oxygen-glucose deprivation (OGD) was reported to activate autophagy and to inhibit mitochondrial fission. At the molecular level, kaempferol reduced Drp1 level. The latter was associated with the reduction of mitochondrial damage and the size of cerebral infarct area in mice undergoing ischemia/reperfusion (Wu et al., 2017). Overall, the results obtained in models of MCAO suggest that autophagy activation in response to ischemia promotes several adaptive mechanisms to counteract the neuronal damage.

The role of autophagy was also investigated in a preclinical model of spontaneous stroke, the stroke-prone spontaneously hypertensive rat (SHRSP). The latter represents a suitable model for studies on human stroke since it shares a multifactorial etiopathogenesis which includes hypertension, genetic and epigenetic factors, and high-salt diet (Rubattu et al., 1996; Stanzione et al., 2020). High salt diet accelerates stroke occurrence in this model. SHRSP rats fed with a high salt stroke-permissive diet failed to activate autophagy/mitophagy at the cerebral level. Restoration of autophagy with the administration of different compounds, such as the synthetic peptide Tat-Beclin1, the natural disaccharide trehalose and nicotinamide mononucleotide, reduced stroke occurrence with a parallel improvement of mitochondrial function (Forte et al., 2020; Forte et al., 2021b). Of interest, reactivation of autophagy rescued endothelial dysfunction in both isolated cerebral endothelial cells and in mesenteric arteries of SHRSP. The latter evidence suggests that vascular autophagy plays a pivotal role in stroke occurrence in the SHRSP rat model. It would be interesting to verify in the future whether an impairment of autophagy occurs also in cerebral cells of SHRSP, such as neurons and astrocytes.

### 5.1 Molecular mechanisms involved in the protective effects of autophagy

The above-described evidence suggests that mTORC1 plays a fundamental role in the inhibition of autophagy in a later phase following ischemia. In fact, mTORC1 inhibition, achieved through rapamycin or by overexpression of TSC-1, was reported to confer beneficial effects. TFEB is also activated in neurons during the first phase of ischemia, where it elicits an adaptive activation of autophagy. In fact, TFEB overexpression reduces ischemic injury in MCAO models. Of interest, the natural disaccharide trehalose was also reported to activate autophagy by targeting TFEB and to reduce stroke occurrence in the SHRSP model (Forte et al., 2021b). Endothelial autophagy appears also to play protective effects in response to cerebral ischemia. However, the molecular mechanisms underlying the effects of endothelial autophagy in the MCAO model have not been fully characterized.

## 6 Detrimental effect of autophagy in animal models of IS

Contrarily to the above discussed evidence, different studies suggest that autophagy plays a detrimental role in few models of IS, leading to neuronal damage (Liang, 2010; Krishnan et al., 2020; Gao et al., 2022). In these cases, brain injury can be counteracted by inhibiting autophagy as demonstrated in the global ischemia/reperfusion rat model (Wang et al., 2011). In fact, autophagy is selectively activated in the damaged hippocampal area from 1 to 48 h of reperfusion after 20-min global ischemia, with a peak at 12 h; in this context, autophagy inhibition by 3-MA, administered before ischemia, prevented the neuronal injury. In contrast, 3-MA was ineffective when administered 60 min following reperfusion (Wang et al., 2011). At the molecular level, increased levels of the lysosomal cysteine protease cathepsin B were detected along with increased necrosis. Consistently, Kim et al. showed that activation of autophagy promoted occludin degradation and contributed to BBB disruption in both brain endothelial cells subjected to OGD and in a rat model of IS (Kim et al., 2020). Brain tissue of rats undergoing permanent focal cerebral ischemia showed autophagy activation in the penumbra area at various times post ischemia (1, 6, 12, 24, 48 h) (Gao et al., 2012). In the same study, the authors evaluated changes in the autophagy process in rats exposed to ischemic post-conditioning (IPOC) and in rats receiving 3-MA administration. Rats undergoing IPOC at the onset of reperfusion showed a reduced cerebral edema and infarct area along with a reduction of ischemia. Interestingly, the neuroprotective effects induced by IPOC were partially reversed by the autophagy inducer rapamycin. Moreover, 3-MA induced a protective effect against focal cerebral ischemia by upregulation of the expression of the antiapoptotic protein Bcl-2 (Gao et al., 2012).

Targeting circ-RNAs may also reduce detrimental autophagy. In this regard, circ_0025984 overexpression reduced cerebral injury in mice undergoing MCAO, along with autophagy inhibition (Zhou et al., 2021). In line with this evidence, circ_016719 expression increased in the brain of mice undergoing MCAO. Moreover, circ_016719 knockdown *in vitro* reduced apoptosis in neurons undergoing oxygen/deprivation along with the reduction of autophagy. However, whether the protective effects of circ_016719 occur through autophagy inhibition should be confirmed by further mechanistic experiments (Tang et al., 2020).

Vitexin, a flavone C-glycoside found in several medical and other plants, reduced brain infarction in the MCAO rat model by suppression of the ischemia-induced autophagy through a mechanism that restored mTOR level and at the same time repressed Ulk1, Beclin1 and the rate of LC3Ⅱ/LC3Ⅰ (Jiang et al., 2018). Consistently, melatonin administration before induction of cerebral ischemia in an I/R mouse model was able to exert protective effects toward brain damage through inhibition of autophagy. Mechanistically, melatonin inhibits endoplasmic reticulum stress-dependent autophagy via protein kinase RNA-like ER kinase (PERK) and inositol-requiring enzyme 1(IRE1) (Feng et al., 2017). When et al. demonstrated that permanent MCAO induced autophagosomes formation from 2 to 12 h after ischemia. Of note, autophagy inhibition by 3-MA or bafilomycin reduced brain edema and motor deficits (Wen et al., 2008). The increase of autophagy was also associated with the increase of cathepsin B. These results suggest that autophagy activation by ischemia may contribute to ischemic neuronal injury. Another study demonstrated that the upregulation of Activin-1 in the peri-infarct region reduced neuronal injury in the MCAO/R mouse model by inhibiting excessive and detrimental autophagy through the PI3K-PKB pathway (Liu et al., 2023). Knockdown of ATG5 in mice undergoing MCAO for 2 h followed by reperfusion for 24 h showed a reduced cerebral infarct area. The latter was associated with reduction of autophagy-related ferroptosis, apoptosis and reactive oxygen species (Zhu et al., 2022). Finally, the administration of netrin-1, a laminin like protein, alleviated ischemic brain damage and improved neurons viability by inhibiting autophagy via PI3K/mTOR pathway both in MCAO rat model and in OGD-rat primary cortical neuronal culture (Tang et al., 2019). Zhang et al. demonstrated that autophagy increased with reperfusion time in mice undergoing transient MCAO and persisted until 24 h. In the same study, the authors found that inhibition of autophagy by 3-MA before ischemia reduced infarct size in mice subjected to permanent MCAO whereas inhibition during reperfusion exacerbated injury. Moreover, knockdown of PARK2, a fundamental mediator of mitophagy also aggravated I/R injury, due to an impaired clearance of damaged mitochondria, which in turn induces cytochrome c release leading to apoptosis. The latter evidence suggests a protective role of autophagy activation during reperfusion (Zhang et al., 2013).

### 6.1 Molecular mechanisms involved in the detrimental effects of autophagy

Excessive levels of autophagy in models of MCAO promote cell death mechanisms, such as apoptosis, necrosis and ferroptosis. Autophagy is also activated by endoplasmic reticulum stress, which in turn contributes to inhibition of the anti-apoptotic factor Bcl-2 (Feng et al., 2017). Because of Bcl-2 inhibition, a detrimental autophagy is activated since Bcl-2 inhibits autophagy by interacting with Beclin-1 (Maiuri et al., 2007). In addition, the reduced levels of Bcl-2 may also explain the increase of apoptosis, since the pro-apoptotic Bax is not inhibited by Bcl-2 (Marquez and Xu, 2012). In the context of detrimental autophagy, the activities of PI3K and mTOR were also inhibited (Tang et al., 2019).

## 7 Autophagy and human IS

Few studies investigated the role of autophagy in the pathogenesis of IS in humans. A genome-wide association study (GWAS) highlighted a significant correlation between three SNPs of ATG7 gene (rs2594966, rs2594973, rs4684776) and the occurrence of stroke due to small-vessel occlusion (SVO) in a cohort of 342 patients and 1,731 controls from the Han Chinese population (Dikic and Elazar, 2018). A significant reduction of autophagy was observed in platelets isolated from smoker subjects affected by metabolic syndrome (MetS) and atrial fibrillation (AF), all conditions known to be associated with a greater risk of developing cardiovascular events including stroke (Carnevale et al., 2021). The reduction of autophagy was associated with increased oxidative stress and platelet aggregation. In the same study the authors demonstrated that the reactivation of autophagy *in vitro* with natural compounds such as trehalose, spermidine and nicotinamide reduced platelet aggregation and oxidative stress (Carnevale et al., 2021). A recent study also demonstrated that serum ATG5 levels correlated with disease progression in patients with IS, suggesting that autophagy level may represent a valid prognostic marker. Subjects affected by IS showed higher serum levels of ATG5 compared to the control group (Ajoolabady et al., 2022). Another report suggests that autophagy inhibition may correlate with stroke occurrence in humans. The thymine T) allele variant at the NADH:Ubiquinone Oxidoreductase Subunit C2 (NDUFC2)/rs11237379, a gene associated with increased occurrence of juvenile IS, correlates with reduced expression of the protein Ndufc2, a subunit of mitochondrial Complex I, causing mitochondrial dysfunction. Endothelial progenitor cells (EPCs) isolated from subjects carrying the T allele failed to activate autophagy in response to stress and showed increased senescence when compared to EPC isolated from wild type individuals at this variant (Rubattu et al., 2016; Forte et al., 2020). The EPC senescence was rescued by Tat-Beclin one treatment (Forte et al., 2020).

### 7.1 Molecular mechanisms underlying autophagy in human IS

Since much of the supporting evidence on the role of autophagy in IS originated from animal studies, human confirmation is needed to support the experimental findings. In this regard, studies performed either in patients with stroke or in subjects at high risk for stroke have provided so far only partial confirmation of the crosstalk between autophagy and the molecular mechanisms highlighted in preclinical models. In the specific, a genome-wide association study revealed that variants falling within the ATG7 gene associated with the occurrence of small-vessel IS, suggesting that autophagosome formation may be defective in human IS (Lee et al., 2017; Dikic and Elazar, 2018). Other evidence suggests that complex I deficiency-dependent mitochondrial dysfunction, along with a reduced clearance of damaged mitochondria by mitophagy, may represent an important contributor to IS in patients (Rubattu et al., 2016; Forte et al., 2020). In fact, a defective Ndufc2 mitochondrial complex I subunit was shown to contribute to an increased risk of juvenile IS in a Caucasian population (Rubattu et al., 2016). More investigation is needed to explore the molecular mechanisms underlying autophagy in the human disease, and, particularly, to dissect out the contribution of both adaptive and maladaptive autophagy in the determination of brain injury in humans.

## 8 Autophagy as therapeutic target in IS

Restoration of autophagy in models of IS in most cases exerts beneficial effects and reduces brain injury. To date, several strategies have been developed to enhance autophagy with pharmacological agents. Natural compounds able to activate autophagy are promising tools, with limited side effects (Frati et al., 2018; Carnevale et al., 2021). Among natural activators of autophagy, the disaccharide trehalose improves vascular function and reduces stroke occurrence in the SHRSP (Forte et al., 2021b). Spermidine, a natural polyamine, reduces platelet aggregation in patients at high risk (Carnevale et al., 2021). To the best of our knowledge, no clinical trials tested the effects of these compounds as an adjuvant therapy to improve stroke recovery or to protect patients at high risk. Synthetic compounds able to activate autophagy have also been developed. In this regard, the Tat-Beclin one is a synthetic peptide able to induce autophagy without targeting other pathways (Shoji-Kawata et al., 2013). Tat-Beclin one was shown to reduce stroke occurrence in the SHRSP and to exert cardiac protective effects (Forte et al., 2020; Forte et al., 2023). However, given its strong effects in the activation of autophagy, this peptide may also induce maladaptive autophagy, leading to autosis, as observed in some circumstances (Nah et al., 2020). Finally, lifestyle modifications, such as intermittent fasting (IF) was also suggested to increase neuronal autophagy and to reduce ageing-related diseases through autophagy dependent mechanisms (Vemuganti and Arumugam, 2021). The latter evidence suggests that IF may represent a potential tool to prevent stroke occurrence in subjects at high risk. Some data also demonstrated that IF decreased cerebral injury in mice undergoing MCAO followed by reperfusion through the reduction of inflammasome (Fann et al., 2014). However, the involvement of autophagy in mediating the protective effects of IF in models of IS needs to be further characterized.

## 9 Conclusion and future perspectives

**FIGURE 2:**
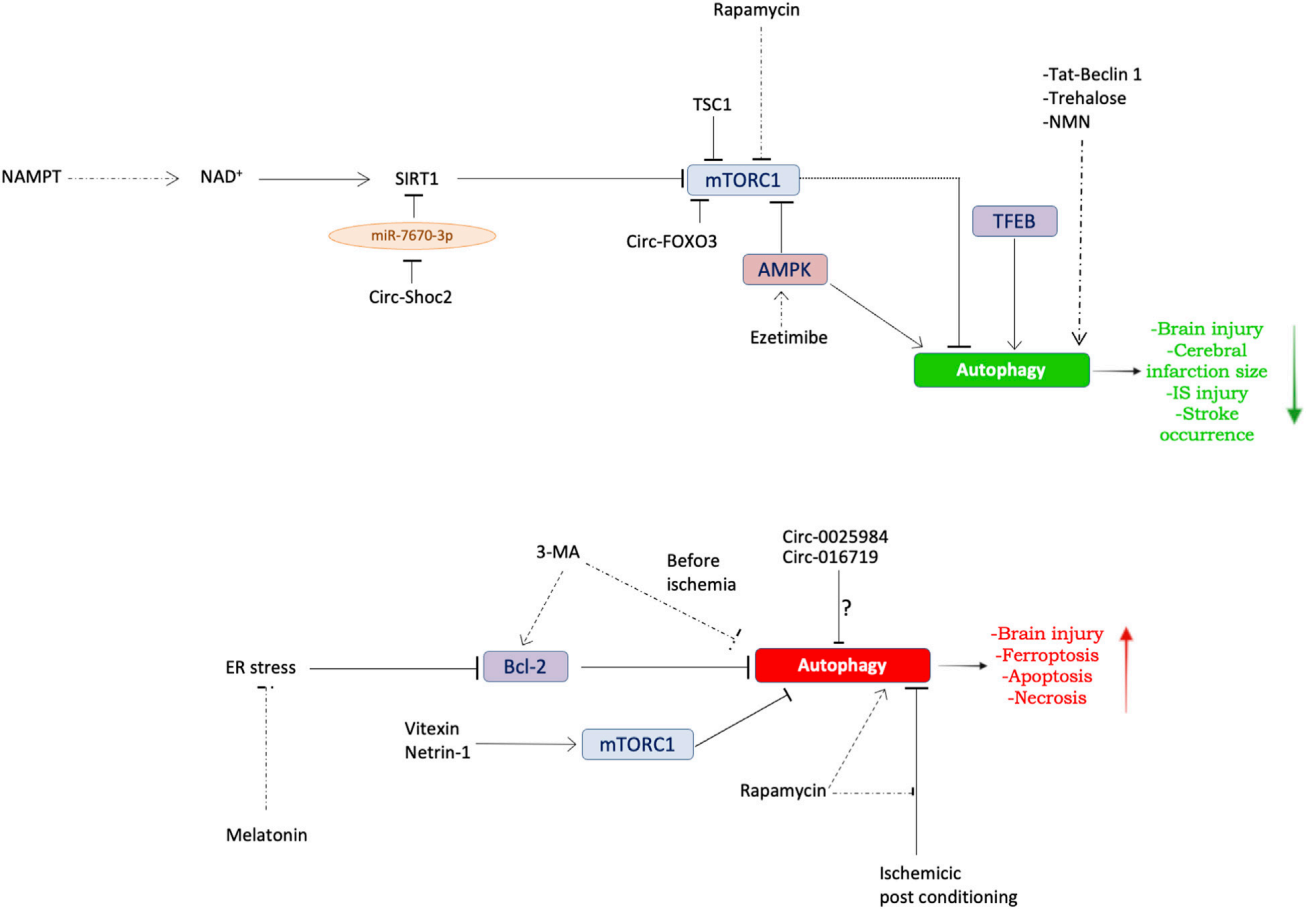
Dual role of autophagy in IS. Schematic overview summarizing the molecular mechanisms underlying autophagy modulation in models of IS. Top panel: evidence regarding the protective role of autophagy in preclinical models of stroke. Bottom panel: evidence regarding the detrimental role of autophagy in preclinical models of stroke. See text for further details. The dotted arrows indicate the exogenous modulation of autophagy.

The evidence discussed herein suggests that autophagy contributes to the pathogenesis of IS (Table 1) (Figure 2). However, a clear comprehension of the exact role played by this molecular mechanism in IS has still to be achieved. The current evidence supports both protective and detrimental effects depending on degree of autophagy activation (Zhang et al., 2013; Forte et al., 2020; Forte et al., 2021a; Forte et al., 2021b). For a better definition of the contribution of autophagy to IS, further efforts could be directed toward the understanding of the involvement of selective forms of autophagy such as mitophagy.

**TABLE 1:** Relevant studies underlying the dual role of autophagy in models of IS.

Protective effects of autophagy in animal models of IS				
Study model	Mechanisms	Effects	Outcomes	References
tMCAO rat	NAMPT overexpression before surgery	↑Autophagy	↓infarct size	Wang et al. (2012)
tMCAO mouse	BAG3 overexpression	↑Autophagy	↓apoptosis	Liu et al. (2023b)
			↓cerebral injury	
pMCAO rat	CAPN1 inhibition	↑autophagic flux	↓ischemic injury	Liu et al. (2021)
pMCAO rat	TFEB overexpression	↑Autophagy	↓ischemic injury	Liu et al. (2019)
tMCAO mouse	Circ-FoxO3 overexpression before surgery	↑Autophagy	↓mTORC1	Yang et al. (2022)
			↑BBB integrity	
tMCAO mouse	circ-SHOC2 overexpression	↑Autophagy	↓brain injury	Chen et al. (2020)
H/I mouse	Rapamycin administration	↑Autophagy	↓mTORC1	Carloni et al. (2008)
			↓necrosis of neurons	
			↓brain injury	
tMCAO rat	Rapamycin administration	↑Autophagy	↓mTORC1	Wu et al. (2018)
			↓infarct size	
			↑neuronal functions	
tMCAO rat	Rapamycin administration	↑Mitophagy	↓mitochondrial dysfunction	Li et al. (2014)
			↓brain injury	
tMCAO rat	Ezetimide administration	↑Autophagy	↓NPC1L1	Yu et al. (2018)
			↓apoptosis	
			↓neuronal damage	
Global forebrain ischemia rat	Tsc1 overexpression	↑Autophagy	↑locomotor activity ↑resistance to ischemia	Lindholm et al. (1987)
I/R mouse	Kaempferol administration	↑Autophagy	↓Drp1	Wu et al. (2017)
			↓mitochondrial fission	
			↓mitochondrial damage	
			↓infarct size	
High salt fed-SHRSP rat	Tat-Beclin, Trehalose and NMN administration	↑Autophagy	↑mitochondrial function	Forte et al. (2020), Forte et al. (2021b)
			↓stroke occurrence	
tMCAO	3-MA during reperfusion	↓Autophagy	↑IR/injury	Zhang et al. (2013)
Detrimental effect of autophagy in animal models of IS				
Study model	Mechanisms	Effects	Outcomes	References
Global I/R rat	3-MA administration 60′ before ischemia	↓Autophagy	↓neuronal necrosis	Wang et al. (2011)
			↓ischemic damage	
pMCAO rat	Occludin degradation	↑Autophagy	↑cellular dysfunction	Kim et al. (2020)
			↓BBB integrity	
pMCAO rat	3-MA administration before reperfusion Ischemic preconditioning	↓Autophagy	↑ischemic damage	Gao et al. (2012)
			↓cerebral edema	
			↓infarct area	
			↓ischemia	
pMCAO mouse	circ_0025984 overexpression	↓Autophagy	↓cerebral injury	Zhou et al. (2021)
tMCAO mouse OGD-mouse neurons cultures	circ_016719 overexpression circ_016719 knockdown	↑Autophagy ↓Autophagy	↑infarct volume	Tang et al. (2020)
			↑apoptosis	
			↓apoptosis	
tMCAO rat	Vitexin administration 1 h before surgery	↓Autophagy	↑mTOR	Jiang et al. (2018)
			↓Ulk1, Beclin1	
			↓LC3II/LC3I	
			↓brain infarction	
I/R mouse	Melatonin administration before ischemia	↓Autophagy	↓PERK	Feng et al. (2017)
			↓IRE1	
			↓brain damage	
pMCAO rat	3-MA/bafilomycin immediately after ischemia	↓Autophagy	↓cathepsin B	Wen et al. (2008)
			↓brain edema	
			↓motor deficits	
tMCAO mouse	Activin-1 administration 6 h after ischemia	↓Autophagy	↓neuronal injury	Liu et al. (2023a)
tMCAO mouse	ATG5 dowregulation before ischemia	↓Autophagy	↓infarct area	Zhu et al. (2024)
			↓ferropoptosis	
			↓apoptosis	
			↓reactive oxygen species	
pMCAO rat	Netrin-1 administration 2 h after surgery	↓Autophagy	↓PI3K/mTOR	Tang et al. (2019)
			↓brain damage	
			↑neurons viability	
pMCAO	3-MA administration before ischemia	↓Autophagy	↓infarct size	Zhang et al. (2013)

Deepening our knowledge on the molecular mechanisms of autophagy and related signal transduction pathways could be useful to avoid the activation of harmful forms of autophagy. Finally, translation of data derived from preclinical studies to the human disease needs to be further developed by both genetic approaches targeting variants of autophagy genes, and by the identification of circulating autophagy biomarkers able to predict stroke predisposition and prognosis. By working on these multiple aspects, we may be able to ultimately develop therapeutic strategies targeting autophagy for the treatment of human IS.

## References

[B1] Ajoolabady A. Shademan B. Avci C. B. Nikanfar M. Nourazarian A. Laghousi D. (2022). Diagnostic potential of autophagy-5 protein, apolipoprotein B-48, and oxidative stress markers in serum of patients with early-stage ischemic stroke. World Neurosurg. 167, e656–e663. 10.1016/j.wneu.2022.08.063 36030010

[B2] Anderson C. M. Macleod K. F. (2019). Autophagy and cancer cell metabolism. Int. Rev. Cell Mol. Biol. 347, 145–190. 10.1016/bs.ircmb.2019.06.002 31451213 PMC8211395

[B3] Balin B. Abrams J. T. Schrogie J. (2011). Toward a unifying hypothesis in the development of Alzheimer's disease. CNS Neurosci. Ther. 17 (6), 587–589. 10.1111/j.1755-5949.2011.00269.x 22117798 PMC6493821

[B4] Benjamin E. J. Muntner P. Alonso A. Bittencourt M. S. Callaway C. W. Carson A. P. (2019). Heart disease and stroke statistics-2019 update: a report from the American heart association. Circulation 139 (10), e56–e528. 10.1161/CIR.0000000000000659 30700139

[B5] Carloni S. Buonocore G. Balduini W. (2008). Protective role of autophagy in neonatal hypoxia-ischemia induced brain injury. Neurobiol. Dis. 32 (3), 329–339. 10.1016/j.nbd.2008.07.022 18760364

[B6] Carnevale R. Nocella C. Schiavon S. Cammisotto V. Cotugno M. Forte M. (2021). Beneficial effects of a combination of natural product activators of autophagy on endothelial cells and platelets. Br. J. Pharmacol. 178 (10), 2146–2159. 10.1111/bph.15399 33512008

[B7] Chen C. Gao H. Su X. (2021). Autophagy-related signaling pathways are involved in cancer (Review). Exp. Ther. Med. 22 (1), 710. 10.3892/etm.2021.10142 34007319 PMC8120650

[B8] Chen W. Wang H. Zhu Z. Feng J. Chen L. (2020). Exosome-shuttled circSHOC2 from IPASs regulates neuronal autophagy and ameliorates ischemic brain injury via the miR-7670-3p/SIRT1 Axis. Mol. Ther. Nucleic Acids 22, 657–672. 10.1016/j.omtn.2020.09.027 33230464 PMC7581834

[B9] Chu C. T. (2008). Eaten alive: autophagy and neuronal cell death after hypoxia-ischemia. Am. J. Pathol. 172 (2), 284–287. 10.2353/ajpath.2008.071064 18202199 PMC2312352

[B10] Cuervo A. M. Wong E. (2014). Chaperone-mediated autophagy: roles in disease and aging. Cell Res. 24 (1), 92–104. 10.1038/cr.2013.153 24281265 PMC3879702

[B11] Dai S. H. Chen T. Li X. Yue K. Y. Luo P. Yang L. K. (2017). Sirt3 confers protection against neuronal ischemia by inducing autophagy: involvement of the AMPK-mTOR pathway. Free Radic. Biol. Med. 108, 345–353. 10.1016/j.freeradbiomed.2017.04.005 28396174

[B12] Debnath J. Gammoh N. Ryan K. M. (2023). Autophagy and autophagy-related pathways in cancer. Nat. Rev. Mol. Cell Biol. 24 (8), 560–575. 10.1038/s41580-023-00585-z 36864290 PMC9980873

[B13] Deng Y. Duan R. Ding W. Gu Q. Liu M. Zhou J. (2022). Astrocyte-derived exosomal nicotinamide phosphoribosyltransferase (Nampt) ameliorates ischemic stroke injury by targeting AMPK/mTOR signaling to induce autophagy. Cell Death Dis. 13 (12), 1057. 10.1038/s41419-022-05454-9 36539418 PMC9767935

[B14] Deretic V. Saitoh T. Akira S. (2013). Autophagy in infection, inflammation and immunity. Nat. Rev. Immunol. 13 (10), 722–737. 10.1038/nri3532 24064518 PMC5340150

[B15] Dikic I. Elazar Z. (2018). Mechanism and medical implications of mammalian autophagy. Nat. Rev. Mol. Cell Biol. 19 (6), 349–364. 10.1038/s41580-018-0003-4 29618831

[B16] Fann D. Y. Santro T. Manzanero S. Widiapradja A. Cheng Y. L. Lee S. Y. (2014). Intermittent fasting attenuates inflammasome activity in ischemic stroke. Exp. Neurol. 257, 114–119. 10.1016/j.expneurol.2014.04.017 24805069

[B17] Favate A. S. Younger D. S. (2016). Epidemiology of ischemic stroke. Neurol. Clin. 34 (4), 967–980. 10.1016/j.ncl.2016.06.013 27720004

[B18] Feng D. Wang B. Wang L. Abraham N. Tao K. Huang L. (2017). Pre-ischemia melatonin treatment alleviated acute neuronal injury after ischemic stroke by inhibiting endoplasmic reticulum stress-dependent autophagy via PERK and IRE1 signalings. J. Pineal Res. 62 (3). 10.1111/jpi.12395 28178380

[B19] Feng J. Chen X. Guan B. Li C. Qiu J. Shen J. (2018). Inhibition of peroxynitrite-induced mitophagy activation attenuates cerebral ischemia-reperfusion injury. Mol. Neurobiol. 55 (8), 6369–6386. 10.1007/s12035-017-0859-x 29307080

[B20] Forte M. Bianchi F. Cotugno M. Marchitti S. De Falco E. Raffa S. (2020). Pharmacological restoration of autophagy reduces hypertension-related stroke occurrence. Autophagy 16 (8), 1468–1481. 10.1080/15548627.2019.1687215 31679456 PMC7469607

[B21] Forte M. Bianchi F. Cotugno M. Marchitti S. Stanzione R. Maglione V. (2021a). An interplay between UCP2 and ROS protects cells from high-salt-induced injury through autophagy stimulation. Cell Death Dis. 12 (10), 919. 10.1038/s41419-021-04188-4 34625529 PMC8501098

[B22] Forte M. Marchitti S. Cotugno M. Di Nonno F. Stanzione R. Bianchi F. (2021b). Trehalose, a natural disaccharide, reduces stroke occurrence in the stroke-prone spontaneously hypertensive rat. Pharmacol. Res. 173, 105875. 10.1016/j.phrs.2021.105875 34500062

[B23] Forte M. Marchitti S. Di Nonno F. Stanzione R. Schirone L. Cotugno M. (2023). NPPA/atrial natriuretic peptide is an extracellular modulator of autophagy in the heart. Autophagy 19 (4), 1087–1099. 10.1080/15548627.2022.2115675 35998113 PMC10012953

[B24] Frati G. Vecchione C. Sciarretta S. (2018). Novel beneficial cardiovascular effects of natural activators of autophagy. Circ. Res. 123 (8), 947–949. 10.1161/CIRCRESAHA.118.313530 30355035

[B25] Gao L. Jiang T. Guo J. Liu Y. Cui G. Gu L. (2012). Inhibition of autophagy contributes to ischemic postconditioning-induced neuroprotection against focal cerebral ischemia in rats. PLoS One 7 (9), e46092. 10.1371/journal.pone.0046092 23029398 PMC3461004

[B26] Gao Y. Wang C. Jiang D. An G. Jin F. Zhang J. (2022). New insights into the interplay between autophagy and oxidative and endoplasmic reticulum stress in neuronal cell death and survival. Front. Cell Dev. Biol. 10, 994037. 10.3389/fcell.2022.994037 36187470 PMC9524158

[B27] Garcia D. Shaw R. J. (2017). AMPK: mechanisms of cellular energy sensing and restoration of metabolic balance. Mol. Cell 66 (6), 789–800. 10.1016/j.molcel.2017.05.032 28622524 PMC5553560

[B28] George P. M. Steinberg G. K. (2015). Novel stroke therapeutics: unraveling stroke pathophysiology and its impact on clinical treatments. Neuron 87 (2), 297–309. 10.1016/j.neuron.2015.05.041 26182415 PMC4911814

[B29] Gomez-Virgilio L. Silva-Lucero M. D. Flores-Morelos D. S. Gallardo-Nieto J. Lopez-Toledo G. Abarca-Fernandez A. M. (2022). Autophagy: a key regulator of homeostasis and disease: an overview of molecular mechanisms and modulators. Cells 11 (15), 2262. 10.3390/cells11152262 35892559 PMC9329718

[B30] Graef M. (2018). Membrane tethering by the autophagy ATG2A-WIPI4 complex. Proc. Natl. Acad. Sci. U. S. A. 115 (42), 10540–10541. 10.1073/pnas.1814759115 30275332 PMC6196487

[B31] Griffey C. J. Yamamoto A. (2022). Macroautophagy in CNS health and disease. Nat. Rev. Neurosci. 23 (7), 411–427. 10.1038/s41583-022-00588-3 35505254 PMC9282724

[B32] Grosjean I. Roméo B. Domdom M. A. Belaid A. D'Andréa G. Guillot N. (2022). Autophagopathies: from autophagy gene polymorphisms to precision medicine for human diseases. Autophagy 18 (11), 2519–2536. 10.1080/15548627.2022.2039994 35383530 PMC9629091

[B33] He J. Liu J. Huang Y. Tang X. Xiao H. Hu Z. (2021). Oxidative stress, inflammation, and autophagy: potential targets of mesenchymal stem cells-based therapies in ischemic stroke. Front. Neurosci. 15, 641157. 10.3389/fnins.2021.641157 33716657 PMC7952613

[B34] Hu Z. Yang B. Mo X. Xiao H. (2015). Mechanism and regulation of autophagy and its role in neuronal diseases. Mol. Neurobiol. 52 (3), 1190–1209. 10.1007/s12035-014-8921-4 25316381

[B35] Jiang J. Dai J. Cui H. (2018). Vitexin reverses the autophagy dysfunction to attenuate MCAO-induced cerebral ischemic stroke via mTOR/Ulk1 pathway. Biomed. Pharmacother. 99, 583–590. 10.1016/j.biopha.2018.01.067 29710456

[B36] Kim K. A. Kim D. Kim J. H. Shin Y. J. Kim E. S. Akram M. (2020). Autophagy-mediated occludin degradation contributes to blood-brain barrier disruption during ischemia in bEnd.3 brain endothelial cells and rat ischemic stroke models. Fluids Barriers CNS 17 (1), 21. 10.1186/s12987-020-00182-8 32169114 PMC7071658

[B37] Klionsky D. J. (2005). The correct way to monitor autophagy in higher eukaryotes. Autophagy 1 (2), 65. 10.4161/auto.1.2.1899 16874029

[B38] Klionsky D. J. Petroni G. Amaravadi R. K. Baehrecke E. H. Ballabio A. Boya P. (2021). Autophagy in major human diseases. EMBO J. 40 (19), e108863. 10.15252/embj.2021108863 34459017 PMC8488577

[B39] Krishnan S. Shrestha Y. Jayatunga D. P. W. Rea S. Martins R. Bharadwaj P. (2020). Activate or inhibit? Implications of autophagy modulation as a therapeutic strategy for Alzheimer's disease. Int. J. Mol. Sci. 21 (18), 6739. 10.3390/ijms21186739 32937909 PMC7554997

[B40] Laplante M. Sabatini D. M. (2013). Regulation of mTORC1 and its impact on gene expression at a glance. J. Cell Sci. 126 (Pt 8), 1713–1719. 10.1242/jcs.125773 23641065 PMC3678406

[B103] Lee T. H. Ko T. M. Chen C. H. Chang Y. J. Lu L. S. Chang C. H. (2017). A genome-wide association study links small-vessel ischemic stroke to autophagy. Sci. Rep. 7, 15229. 29123153 10.1038/s41598-017-14355-3 PMC5680343

[B41] Levine B. Kroemer G. (2019). Biological functions of autophagy genes: a disease perspective. Cell 176 (1-2), 11–42. 10.1016/j.cell.2018.09.048 30633901 PMC6347410

[B42] Li L. Li L. Zhou X. Yu Y. Li Z. Zuo D. (2019). Silver nanoparticles induce protective autophagy via Ca2+/CaMKKβ/AMPK/mTOR pathway in SH-SY5Y cells and rat brains. Nanotoxicology 13 (3), 369–391. 10.1080/17435390.2018.1550226 30729847

[B43] Li Q. Zhang T. Wang J. Zhang Z. Zhai Y. Yang G. Y. (2014). Rapamycin attenuates mitochondrial dysfunction via activation of mitophagy in experimental ischemic stroke. Biochem. Biophys. Res. Commun. 444 (2), 182–188. 10.1016/j.bbrc.2014.01.032 24440703

[B44] Li W. L. Yu S. P. Chen D. Yu S. S. Jiang Y. J. Genetta T. (2013). The regulatory role of NF-κB in autophagy-like cell death after focal cerebral ischemia in mice. Neuroscience 244, 16–30. 10.1016/j.neuroscience.2013.03.045 23558089 PMC3916093

[B45] Li W. W. Li J. Bao J. K. (2012). Microautophagy: lesser-known self-eating. Cell Mol. Life Sci. 69 (7), 1125–1136. 10.1007/s00018-011-0865-5 22080117 PMC11114512

[B46] Li X. Li L. Si X. Zhang Z. Ni Z. Zhou Y. (2022). The regulatory roles of circular RNAs via autophagy in ischemic stroke. Front. Neurol. 13, 963508. 10.3389/fneur.2022.963508 36330428 PMC9623297

[B47] Liang C. (2010). Negative regulation of autophagy. Cell Death Differ. 17 (12), 1807–1815. 10.1038/cdd.2010.115 20865012 PMC3131090

[B48] Liao X. Sluimer J. C. Wang Y. Subramanian M. Brown K. Pattison J. S. (2012). Macrophage autophagy plays a protective role in advanced atherosclerosis. Cell Metab. 15 (4), 545–553. 10.1016/j.cmet.2012.01.022 22445600 PMC3322248

[B49] Lindholm L. Mobacken H. Magnusson B. L. (1987). Circulating immune complexes in untreated psoriasis. A comparison of Raji-cell radioimmunoassay and polymorphonuclear leukocyte phagocytosis. Arch. Dermatol Res. 279 (7), 435–438. 10.1007/BF00412587 3435171

[B50] Liu M. Li Y. Han S. Wang H. Li J. (2023a). Activin A alleviates neuronal injury through inhibiting cGAS-STING-mediated autophagy in mice with ischemic stroke. J. Cereb. Blood Flow. Metab. 43 (5), 736–748. 10.1177/0271678X221147056 36537048 PMC10108189

[B51] Liu X. Ye Q. Huang Z. Li X. Zhang L. Liu X. (2023b). BAG3 overexpression attenuates ischemic stroke injury by activating autophagy and inhibiting apoptosis. Stroke 54 (8), 2114–2125. 10.1161/STROKEAHA.123.041783 37377010

[B52] Liu Y. Che X. Zhang H. Fu X. Yao Y. Luo J. (2021). CAPN1 (Calpain1)-Mediated impairment of autophagic flux contributes to cerebral ischemia-induced neuronal damage. Stroke 52 (5), 1809–1821. 10.1161/STROKEAHA.120.032749 33874744

[B53] Liu Y. Xue X. Zhang H. Che X. Luo J. Wang P. (2019). Neuronal-targeted TFEB rescues dysfunction of the autophagy-lysosomal pathway and alleviates ischemic injury in permanent cerebral ischemia. Autophagy 15 (3), 493–509. 10.1080/15548627.2018.1531196 30304977 PMC6351122

[B54] Ma Y. Galluzzi L. Zitvogel L. Kroemer G. (2013). Autophagy and cellular immune responses. Immunity 39 (2), 211–227. 10.1016/j.immuni.2013.07.017 23973220

[B55] Maiuri M. C. Le Toumelin G. Criollo A. Rain J. C. Gautier F. Juin P. (2007). Functional and physical interaction between Bcl-X(L) and a BH3-like domain in Beclin-1. EMBO J. 26 (10), 2527–2539. 10.1038/sj.emboj.7601689 17446862 PMC1868901

[B56] Marino G. Pietrocola F. Madeo F. Kroemer G. (2014). Caloric restriction mimetics: natural/physiological pharmacological autophagy inducers. Autophagy 10 (11), 1879–1882. 10.4161/auto.36413 25484097 PMC4502795

[B57] Marquez R. T. Xu L. (2012). Bcl-2:Beclin 1 complex: multiple, mechanisms regulating autophagy/apoptosis toggle switch. Am. J. Cancer Res. 2 (2), 214–221. 22485198 PMC3304572

[B58] Matoba K. Kotani T. Tsutsumi A. Tsuji T. Mori T. Noshiro D. (2020). Atg9 is a lipid scramblase that mediates autophagosomal membrane expansion. Nat. Struct. Mol. Biol. 27 (12), 1185–1193. 10.1038/s41594-020-00518-w 33106658

[B59] Medeiros R. Baglietto-Vargas D. LaFerla F. M. (2011). The role of tau in Alzheimer's disease and related disorders. CNS Neurosci. Ther. 17 (5), 514–524. 10.1111/j.1755-5949.2010.00177.x 20553310 PMC4072215

[B60] Mehta S. L. Pandi G. Vemuganti R. (2017). Circular RNA expression profiles alter significantly in mouse brain after transient focal ischemia. Stroke 48 (9), 2541–2548. 10.1161/STROKEAHA.117.017469 28701578 PMC5575968

[B61] Menikdiwela K. R. Ramalingam L. Rasha F. Wang S. Dufour J. M. Kalupahana N. S. (2020). Autophagy in metabolic syndrome: breaking the wheel by targeting the renin-angiotensin system. Cell Death Dis. 11 (2), 87. 10.1038/s41419-020-2275-9 32015340 PMC6997396

[B62] Nah J. Zhai P. Huang C. Y. Fernandez A. F. Mareedu S. Levine B. (2020). Upregulation of Rubicon promotes autosis during myocardial ischemia/reperfusion injury. J. Clin. Invest. 130 (6), 2978–2991. 10.1172/JCI132366 32364533 PMC7260042

[B63] Nishimura T. Kaizuka T. Cadwell K. Sahani M. H. Saitoh T. Akira S. (2013). FIP200 regulates targeting of Atg16L1 to the isolation membrane. EMBO Rep. 14 (3), 284–291. 10.1038/embor.2013.6 23392225 PMC3589088

[B64] Nixon R. A. (2013). The role of autophagy in neurodegenerative disease. Nat. Med. 19 (8), 983–997. 10.1038/nm.3232 23921753

[B65] Papadakis M. Hadley G. Xilouri M. Hoyte L. C. Nagel S. McMenamin M. M. (2013). Tsc1 (hamartin) confers neuroprotection against ischemia by inducing autophagy. Nat. Med. 19 (3), 351–357. 10.1038/nm.3097 23435171 PMC3744134

[B66] Perez-Alvarez M. J. Villa Gonzalez M. Benito-Cuesta I. Wandosell F. G. (2018). Role of mTORC1 controlling proteostasis after brain ischemia. Front. Neurosci. 12, 60. 10.3389/fnins.2018.00060 29497356 PMC5818460

[B67] Pickford F. Masliah E. Britschgi M. Lucin K. Narasimhan R. Jaeger P. A. (2008). The autophagy-related protein beclin 1 shows reduced expression in early Alzheimer disease and regulates amyloid beta accumulation in mice. J. Clin. Invest. 118 (6), 2190–2199. 10.1172/JCI33585 18497889 PMC2391284

[B68] Qi Z. Dong W. Shi W. Wang R. Zhang C. Zhao Y. (2015). Bcl-2 phosphorylation triggers autophagy switch and reduces mitochondrial damage in limb remote ischemic conditioned rats after ischemic stroke. Transl. Stroke Res. 6 (3), 198–206. 10.1007/s12975-015-0393-y 25744447

[B69] Rami A. (2008). Upregulation of Beclin 1 in the ischemic penumbra. Autophagy 4 (2), 227–229. 10.4161/auto.5339 18075295

[B70] Rana A. K. Singh D. (2018). Targeting glycogen synthase kinase-3 for oxidative stress and neuroinflammation: opportunities, challenges and future directions for cerebral stroke management. Neuropharmacology 139, 124–136. 10.1016/j.neuropharm.2018.07.006 30017999

[B71] Rubattu S. Di Castro S. Schulz H. Geurts A. M. Cotugno M. Bianchi F. (2016). Ndufc2 gene inhibition is associated with mitochondrial dysfunction and increased stroke susceptibility in an animal model of complex human disease. J. Am. Heart Assoc. 5 (2), e002701. 10.1161/JAHA.115.002701 26888427 PMC4802485

[B72] Rubattu S. Volpe M. Kreutz R. Ganten U. Ganten D. Lindpaintner K. (1996). Chromosomal mapping of quantitative trait loci contributing to stroke in a rat model of complex human disease. Nat. Genet. 13 (4), 429–434. 10.1038/ng0896-429 8696337

[B73] Sciarretta S. Maejima Y. Zablocki D. Sadoshima J. (2018). The role of autophagy in the heart. Annu. Rev. Physiol. 80, 1–26. 10.1146/annurev-physiol-021317-121427 29068766

[B74] Shi G. S. Qin Q. L. Huang C. Li Z. R. Wang Z. H. Wang Y. Y. (2023). The pathological mechanism of neuronal autophagy-lysosome dysfunction after ischemic stroke. Cell Mol. Neurobiol. 43 (7), 3251–3263. 10.1007/s10571-023-01382-0 37382853 PMC11410006

[B75] Shoji-Kawata S. Sumpter R. Leveno M. Campbell G. R. Zou Z. Kinch L. (2013). Identification of a candidate therapeutic autophagy-inducing peptide. Nature 494 (7436), 201–206. 10.1038/nature11866 23364696 PMC3788641

[B76] Stanzione R. Cotugno M. Bianchi F. Marchitti S. Forte M. Volpe M. (2020). Pathogenesis of ischemic stroke: role of epigenetic mechanisms. Genes (Basel) 11 (1), 89. 10.3390/genes11010089 31941075 PMC7017187

[B77] Tang C. Ou J. Kou L. Deng J. Luo S. (2020). Circ_016719 plays a critical role in neuron cell apoptosis induced by I/R via targeting miR-29c/Map2k6. Mol. Cell Probes 49, 101478. 10.1016/j.mcp.2019.101478 31698040

[B78] Tang T. Gao D. Yang X. Hua X. Li S. Sun H. (2019). Exogenous netrin-1 inhibits autophagy of ischemic brain tissues and hypoxic neurons via PI3K/mTOR pathway in ischemic stroke. J. Stroke Cerebrovasc. Dis. 28 (5), 1338–1345. 10.1016/j.jstrokecerebrovasdis.2019.01.032 30797642

[B79] Uchiyama Y. Koike M. Shibata M. (2008). Autophagic neuron death in neonatal brain ischemia/hypoxia. Autophagy 4 (4), 404–408. 10.4161/auto.5598 18212531

[B80] Vemuganti R. Arumugam T. V. (2021). Much ado about eating: intermittent fasting and post-stroke neuroprotection. J. Cereb. Blood Flow. Metab. 41 (7), 1791–1793. 10.1177/0271678X211009362 33853407 PMC8221776

[B81] Wang C. Wang H. Zhang D. Luo W. Liu R. Xu D. (2018a). Phosphorylation of ULK1 affects autophagosome fusion and links chaperone-mediated autophagy to macroautophagy. Nat. Commun. 9 (1), 3492. 10.1038/s41467-018-05449-1 30154410 PMC6113293

[B82] Wang J. Y. Xia Q. Chu K. T. Pan J. Sun L. N. Zeng B. (2011). Severe global cerebral ischemia-induced programmed necrosis of hippocampal CA1 neurons in rat is prevented by 3-methyladenine: a widely used inhibitor of autophagy. J. Neuropathol. Exp. Neurol. 70 (4), 314–322. 10.1097/NEN.0b013e31821352bd 21412169

[B83] Wang P. Guan Y. F. Du H. Zhai Q. W. Su D. F. Miao C. Y. (2012). Induction of autophagy contributes to the neuroprotection of nicotinamide phosphoribosyltransferase in cerebral ischemia. Autophagy 8 (1), 77–87. 10.4161/auto.8.1.18274 22113203

[B84] Wang P. Shao B. Z. Deng Z. Chen S. Yue Z. Miao C. Y. (2018b). Autophagy in ischemic stroke. Prog. Neurobiol. 163-164, 98–117. 10.1016/j.pneurobio.2018.01.001 29331396

[B85] Wang X. Fang Y. Huang Q. Xu P. Lenahan C. Lu J. (2021). An updated review of autophagy in ischemic stroke: from mechanisms to therapies. Exp. Neurol. 340, 113684. 10.1016/j.expneurol.2021.113684 33676918

[B86] Wei H. Li Y. Han S. Liu S. Zhang N. Zhao L. (2016). cPKCγ-modulated autophagy in neurons alleviates ischemic injury in brain of mice with ischemic stroke through akt-mTOR pathway. Transl. Stroke Res. 7 (6), 497–511. 10.1007/s12975-016-0484-4 27510769

[B87] Wen Y. D. Sheng R. Zhang L. S. Han R. Zhang X. Zhang X. D. (2008). Neuronal injury in rat model of permanent focal cerebral ischemia is associated with activation of autophagic and lysosomal pathways. Autophagy 4 (6), 762–769. 10.4161/auto.6412 18567942

[B88] Wesselborg S. Stork B. (2015). Autophagy signal transduction by ATG proteins: from hierarchies to networks. Cell Mol. Life Sci. 72 (24), 4721–4757. 10.1007/s00018-015-2034-8 26390974 PMC4648967

[B89] Williams O. A. Zeestraten E. A. Benjamin P. Lambert C. Lawrence A. J. Mackinnon A. D. (2019). Predicting dementia in cerebral small vessel disease using an automatic diffusion tensor image segmentation technique. Stroke 50 (10), 2775–2782. 10.1161/STROKEAHA.119.025843 31510902 PMC6756294

[B90] Wu B. Luo H. Zhou X. Cheng C. Y. Lin L. Liu B. L. (2017). Succinate-induced neuronal mitochondrial fission and hexokinase II malfunction in ischemic stroke: therapeutical effects of kaempferol. Biochim. Biophys. Acta Mol. Basis Dis. 1863 (9), 2307–2318. 10.1016/j.bbadis.2017.06.011 28634116

[B91] Wu M. Zhang H. Kai J. Zhu F. Dong J. Xu Z. (2018). Rapamycin prevents cerebral stroke by modulating apoptosis and autophagy in penumbra in rats. Ann. Clin. Transl. Neurol. 5 (2), 138–146. 10.1002/acn3.507 29468175 PMC5817831

[B92] Xu D. Kong T. Zhang S. Cheng B. Chen J. Wang C. (2021). Orexin-A protects against cerebral ischemia-reperfusion injury by inhibiting excessive autophagy through OX1R-mediated MAPK/ERK/mTOR pathway. Cell Signal 79, 109839. 10.1016/j.cellsig.2020.109839 33212156

[B93] Yamamoto H. Matsui T. (2023). Molecular mechanisms of macroautophagy, microautophagy, and chaperone-mediated autophagy. J. Nippon. Med. Sch. 91, 2–9. 10.1272/jnms.JNMS.2024_91-102 37271546

[B94] Yang Z. Goronzy J. J. Weyand C. M. (2015). Autophagy in autoimmune disease. J. Mol. Med. Berl. 93 (7), 707–717. 10.1007/s00109-015-1297-8 26054920 PMC4486076

[B95] Yang Z. Huang C. Wen X. Liu W. Huang X. Li Y. (2022). Circular RNA circ-FoxO3 attenuates blood-brain barrier damage by inducing autophagy during ischemia/reperfusion. Mol. Ther. 30 (3), 1275–1287. 10.1016/j.ymthe.2021.11.004 34763084 PMC8899525

[B96] Yu J. Li X. Matei N. McBride D. Tang J. Yan M. (2018). Ezetimibe, a NPC1L1 inhibitor, attenuates neuronal apoptosis through AMPK dependent autophagy activation after MCAO in rats. Exp. Neurol. 307, 12–23. 10.1016/j.expneurol.2018.05.022 29852178

[B97] Zeng L. Hu S. Chen R. Li H. Yu J. Yang H. (2023). Animal models of ischemic stroke with different forms of middle cerebral artery occlusion. Brain Sci. 13 (7), 1007. 10.3390/brainsci13071007 37508939 PMC10377124

[B98] Zhan L. Chen S. Li K. Liang D. Zhu X. Liu L. (2017). Autophagosome maturation mediated by Rab7 contributes to neuroprotection of hypoxic preconditioning against global cerebral ischemia in rats. Cell Death Dis. 8 (7), e2949. 10.1038/cddis.2017.330 28726776 PMC5550874

[B99] Zhang X. Yan H. Yuan Y. Gao J. Shen Z. Cheng Y. (2013). Cerebral ischemia-reperfusion-induced autophagy protects against neuronal injury by mitochondrial clearance. Autophagy 9 (9), 1321–1333. 10.4161/auto.25132 23800795

[B100] Zhou D. Huang Z. Zhu X. Hong T. Zhao Y. (2021). Circular RNA 0025984 ameliorates ischemic stroke injury and protects astrocytes through miR-143-3p/TET1/orp150 pathway. Mol. Neurobiol. 58 (11), 5937–5953. 10.1007/s12035-021-02486-8 34435328

[B101] Zhu H. Zhong Y. Chen R. Wang L. Li Y. Jian Z. (2022). ATG5 knockdown attenuates ischemia‒reperfusion injury by reducing excessive autophagy-induced ferroptosis. Transl. Stroke Res. 15, 153–164. 10.1007/s12975-022-01118-0 36522583

[B102] Zhu H. Zhong Y. Chen R. Wang L. Li Y. Jian Z. (2024). ATG5 knockdown attenuates ischemia‒reperfusion injury by reducing excessive autophagy-induced ferroptosis. Transl. Stroke Res. 15 (1), 153–164. 10.1007/s12975-022-01118-0 36522583

